# Dietary Vitamin K Intake and Insulin Resistance Markers in U.S. Adults: NHANES 2001–2018

**DOI:** 10.3390/jcm15103763

**Published:** 2026-05-14

**Authors:** Wojciech Matuszewski, Mikołaj Madeksza, Michał Szklarz, Paulina Włodarczyk, Patrycja Waśniewska, Judyta Juranek

**Affiliations:** 1 Clinic of Endocrinology and Metabolic Diseases, School of Medicine, Collegium Medicum, University of Warmia and Mazury in Olsztyn, 10-957 Olsztyn, Poland; mikolaj.madeksza@icloud.com (M.M.);; 2 Department of Human Physiology and Pathophysiology, University of Warmia and Mazury in Olsztyn, 10-719 Olsztyn, Poland

**Keywords:** vitamin K, insulin resistance, HOMA-IR, fasting insulin, glucose metabolism, NHANES, type 2 diabetes

## Abstract

**Background:** Vitamin K (VK) has emerging roles beyond coagulation, including potential involvement in glucose metabolism. While large cohorts report inverse associations between VK status and incident type 2 diabetes, less is known about its relationship with insulin resistance markers in general populations. **Methods:** We conducted a cross-sectional analysis of 23,247 adults from the National Health and Nutrition Examination Survey (NHANES) 2001–2018. Dietary VK intake was assessed using a single 24 h recall and modeled as energy-adjusted nutrient density (µg/1000 kcal). Fasting insulin, homeostasis model assessment of insulin resistance (HOMA-IR), fasting glucose, and HbA1c were analyzed using survey-weighted linear regression; models were adjusted for demographic, socioeconomic, lifestyle, and adiposity-related factors. Effect modification by baseline metabolic status was evaluated, and sensitivity analyses assessed robustness. **Results:** Higher energy-adjusted VK intake was modestly associated with lower fasting insulin and HOMA-IR, but not with fasting glucose or HbA1c in primary linear models. Each SD increase in VK intake was associated with 1.4% lower fasting insulin (95% CI: −2.4% to −0.4%) and 1.3% lower HOMA-IR (95% CI: −2.3% to −0.3%) in fully adjusted models. Associations were attenuated following adjustment for adiposity. Effect modification by baseline metabolic status was observed, with stronger associations among normoglycemic individuals. Findings were consistent across sensitivity analyses, although effect sizes were small. **Conclusions:** In this nationally representative sample of U.S. adults, higher VK intake was modestly associated with lower insulin resistance markers. Prospective studies incorporating objective VK biomarkers and dynamic measures of insulin sensitivity are needed to clarify these associations.

## 1. Introduction

Vitamin K (VK), traditionally recognized for its role in coagulation, is increasingly implicated in extrahepatic processes, including metabolic regulation [1,2,3,4]. These extrahepatic roles require a higher VK status than is needed to maintain normal hemostasis, and a substantial proportion of the population is thought to have subclinical VK deficiency—a state in which coagulation is preserved but VK availability may be insufficient to support its broader physiological roles [5,6,7,8,9]. Observational evidence links lower VK status to adverse metabolic outcomes, including insulin resistance and type 2 diabetes (T2D) [10,11,12], greater adiposity [7,13,14,15,16,17], elevated inflammatory markers [7,11,18,19,20,21,22,23], and less favorable lipid profiles [7,11,13,24].

Mechanistic studies have also suggested potential links between VK and glucose metabolism [4]. In pancreatic β-cells, VK has been reported to influence cellular processes such as endoplasmic reticulum calcium handling and cAMP-dependent signaling [25,26]. In peripheral tissues, VK may enhance insulin sensitivity via SIRT1/AMPK activation and anti-inflammatory pathways [27,28,29,30,31,32,33].

Results from supplementation trials remain heterogeneous: effects on fasting glucose are generally limited [4,7,34,35,36,37], whereas findings for insulin sensitivity appear to be more consistent among individuals with prediabetes or diabetes [4,38,39,40].

Taken together, existing evidence suggests a potential relationship between VK and glucose metabolism, but its nature and magnitude in human populations remain uncertain. It is unclear whether observed associations reflect direct biological effects, confounding by dietary or lifestyle factors, or indirect pathways such as adiposity or inflammation. It is also uncertain whether these associations differ across metabolic states. Although recent NHANES analyses have explored associations between VK intake and metabolic outcomes such as dyslipidemia, including insulin resistance-related variables in secondary analyses [24], a direct evaluation of VK intake in relation to glucose metabolism in this large population-based sample is lacking.

Here, we examined the association between dietary VK intake and glycemic markers among adults using data from NHANES 2001–2018. The use of a large, nationally representative sample with standardized laboratory measurements allowed evaluation of these associations at the population level. Additionally, we assessed effect modification by baseline metabolic status and quantified attenuation by adiposity, providing insight into the metabolic contexts in which these associations may differ.

## 2. Materials and Methods

### 2.1. Study Population

**Figure 1:**
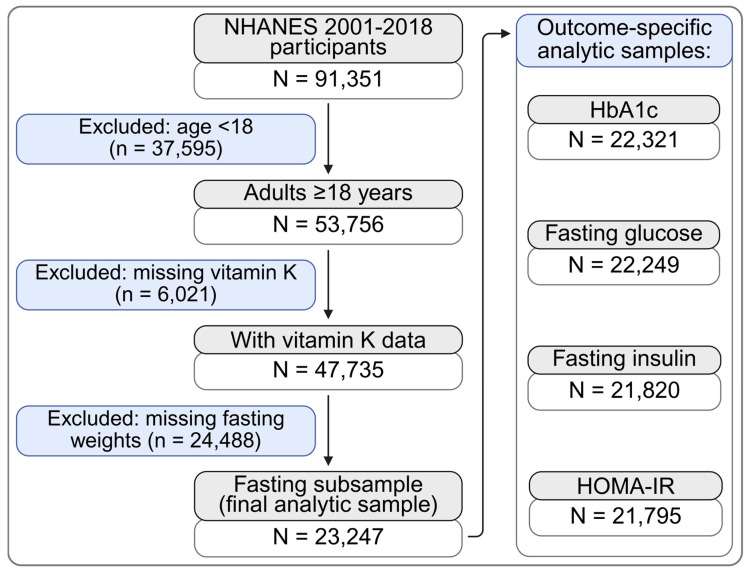
Study population and analytic samples. Notes: The final analytic sample included adults aged ≥18 years with available dietary vitamin K data and fasting subsample weights. Outcome-specific analytic samples reflect complete-case analyses for each glycemic marker and therefore differ in sample size. Created in BioRender. Madeksza, M. (2026). https://BioRender.com/ycvs7r1 (accessed on 4 May 2026).

We analyzed data from the National Health and Nutrition Examination Survey (NHANES). NHANES is a program of cross-sectional studies conducted by the National Center for Health Statistics (NCHS), which assesses the health and nutritional status of a nationally representative sample of the U.S. population. We pooled nine consecutive NHANES cycles (2001–2018) because they included concurrent measurements of dietary VK intake and fasting glycemic markers. Among adults aged ≥18 years, we excluded participants with missing dietary VK data, fasting laboratory measurements, or survey design variables. The final analytic sample included 23,247 adults; sample sizes varied slightly by outcome due to missing laboratory data (Figure 1). NHANES protocols were approved by the NCHS Ethics Review Board, and all participants provided written informed consent [41].

### 2.2. Assessment of Dietary VK Intake

VK intake (µg/day) was assessed using the Day 1 24 h dietary recall collected via the USDA Automated Multiple-Pass Method. VK intake was energy-adjusted using the nutrient density method (µg per 1000 kcal). For primary analyses, it was categorized into survey-weighted quartiles (Q1–Q4), with Q1 representing the lowest intake. For continuous analyses, VK density was modeled per SD increase (1 SD = 102.5 µg/1000 kcal).

### 2.3. Assessment of Glycemic Markers

Blood samples were collected as part of the NHANES examination protocol during the participant’s examination visit after an overnight fast and processed according to standardized NHANES laboratory protocols.

Primary outcomes were fasting insulin and insulin resistance estimated using the homeostasis model assessment of insulin resistance (HOMA-IR), calculated as follows:

HOMA-IR = (fasting insulin (µU/mL) × fasting glucose (mg/dL))/405.

Fasting insulin and HOMA-IR were natural log-transformed in all analyses due to right-skewed distributions. Secondary outcomes included fasting glucose and glycated hemoglobin (HbA1c); they were analyzed on their original scale.

### 2.4. Covariates

We selected covariates based on biological plausibility and prior evidence. These included: age (years, continuous); sex (male/female); race/ethnicity; poverty–income ratio (continuous); smoking status (current/non-current smoker); alcohol use (ever/never drinker); total energy intake (kcal/day); body mass index (BMI, kg/m^2^); and waist circumference (cm).

### 2.5. Statistical Analysis

All analyses followed the NHANES Analytic and Reporting Guidelines and used sampling weights to account for oversampling and the complex, multistage, clustered sampling design. The weighted sample is representative of the U.S. adult population [42]. Because fasting laboratory measurements were available only in a subsample, we used the NHANES fasting subsample weights (WTSAF2YR) for all primary analyses. To account for pooling of nine survey cycles (2001–2018), 2-year fasting weights were divided by the number of cycles combined. For sensitivity analyses restricted to NHANES cycles with available Healthy Eating Index (HEI-2020) data (2005–2018), weights were analogously rescaled by the number of included cycles. Survey design variables for primary sampling units (SDMVPSU) and strata (SDMVSTRA) were incorporated to account for clustering and stratification in the sampling design. All analyses were performed using R (version 4.5.2) with the survey package, which applies Taylor series linearization for variance estimation. Two-sided *p*-values < 0.05 were considered statistically significant.

The full prespecified analysis plan is shown in Supplementary Figure S2. We summarized descriptive statistics across quartiles of energy-adjusted VK intake using weighted percentages for categorical variables and survey-weighted means ± standard deviations (SDs) for continuous variables, except for energy-adjusted VK intake, insulin, and HOMA-IR, which were presented as medians (interquartile ranges) due to right-skewed distributions.

We used survey-weighted linear regression models to examine associations between energy-adjusted VK intake and glycemic markers. VK intake was analyzed in quartiles, with the lowest quartile (Q1) serving as a reference; linear trends were assessed by modeling the median intake within each quartile as a continuous variable. VK intake was also modeled continuously per SD increase in VK density (1 SD = 102.5 µg/1000 kcal). All glycemic markers were modeled as continuous outcomes. For each outcome, analyses were restricted to participants with non-missing values for that specific marker, and all covariates were included in the corresponding model (complete-case analysis). Insulin and HOMA-IR were natural log-transformed, and results are presented as percent differences.

Three models were specified: Model 1 (unadjusted); Model 2, adjusted for age, sex, race/ethnicity, poverty–income ratio, smoking status, alcohol use, and total energy intake; and Model 3, additionally adjusted for BMI and waist circumference. BMI and waist circumference were included simultaneously to account for both overall and central adiposity, allowing for more comprehensive adjustment for body composition. To describe changes in the association after adjustment for adiposity, we calculated percent attenuation as the proportional change in β coefficients between sequential models.

We evaluated potential non-linearity using survey-weighted restricted cubic spline models (3 degrees of freedom), with Wald tests used to assess overall association and non-linearity.

Sensitivity analyses included additional adjustment for overall diet quality (HEI-2020) and vigorous physical activity; exclusions of implausible energy intake, extreme VK intake (1st–99th percentiles), or diabetes; alternative specifications of adiposity (BMI or waist circumference modeled separately); and modeling VK intake as a continuous exposure on the natural logarithmic scale.

We assessed effect modification by testing interaction terms between continuous energy-adjusted VK intake (per SD) and baseline metabolic status (normal vs. impaired glucose metabolism, defined as a fasting glucose level of 100–125 mg/dL or an HbA1c of 5.7–6.4%), obesity status (BMI < 30 kg/m^2^ vs. ≥30 kg/m^2^) and sex. Analyses of baseline metabolic status were restricted to participants without diabetes to reduce potential confounding from disease-related behavioral changes and treatment. Interaction significance was assessed using Wald tests.

## 3. Results

### 3.1. Study Population Characteristics

The analytic sample included 23,247 adults from NHANES 2001–2018 (Figure 1). Survey-weighted characteristics by quartiles of energy-adjusted VK intake are shown in Table 1. Participants in higher VK quartiles were older, more often female, had higher poverty–income ratios, and were less likely to be current smokers. Total energy intake was lower in the highest quartile, whereas BMI and waist circumference were similar across quartiles. The prevalence of diabetes increased slightly across quartiles. Unadjusted fasting insulin and HOMA-IR were lower across increasing VK quartiles, whereas fasting glucose and HbA1c differed modestly.

### 3.2. Association Between Dietary Vitamin K Intake and Glycemic Markers

Associations between energy-adjusted VK intake and glycemic markers are presented in Table 2 and Table 3. In crude models (Model 1), higher VK intake was negatively associated with fasting insulin and HOMA-IR, and it was positively associated with fasting glucose and HbA1c.

After multivariable adjustment, associations with fasting glucose and HbA1c were no longer significant. In contrast, higher VK intake remained modestly associated with lower fasting insulin and HOMA-IR: in fully adjusted models (Model 3), participants in the highest quartile of VK intake had 6.6% lower fasting insulin and 6.2% lower HOMA-IR compared with the lowest quartile (both *p* for trend <0.001).

When modeled continuously (per 1 SD increase in energy-adjusted VK intake), each SD increase in VK intake was associated with a 1.4% lower fasting insulin (95% CI: −2.4% to −0.4%; *p* = 0.0067) and a 1.3% lower HOMA-IR (95% CI: −2.3% to −0.3%; *p* = 0.014). Adjustment for demographic and lifestyle factors modestly attenuated associations (13–19%), while additional adjustment for adiposity accounted for 44–49% of the remaining association (Supplementary Table S4). Findings for fasting glucose and HbA1c remained null.

### 3.3. Effect Modification by Baseline Metabolic Status, Obesity, and Sex

Associations differed significantly by baseline glycemic status (*p* for interaction = 0.0052 for insulin, 0.0062 for HOMA-IR) (Table 4). In normoglycemic participants, each SD increase in energy-adjusted VK intake was associated with a 3.4% lower fasting insulin level (95% CI: −5.2% to −1.6%) and 3.4% lower HOMA-IR (95% CI: −5.2% to −1.5%), whereas in participants with impaired glucose metabolism, associations were attenuated but remained statistically significant (insulin: −0.8%; HOMA-IR: −0.7%).

No significant interactions were observed for obesity status or sex (all *p*-values for interaction > 0.05; Supplementary Table S6).

### 3.4. Sensitivity Analyses

Associations between energy-adjusted VK intake and insulin resistance markers were consistent across sensitivity analyses (Supplementary Tables S1–S3). Additional adjustment for overall diet quality (HEI-2020) moderately attenuated effect sizes, but associations remained statistically significant (insulin: −1.1% vs. −1.4% in the primary analysis; HOMA-IR: −0.9% vs. −1.3%). Adjustment for vigorous physical activity resulted in a modest attenuation (insulin: −1.3%; HOMA-IR: −1.2%). Exclusion of extreme VK intake values strengthened the associations (insulin: −2.3%; HOMA-IR: −2.2%), whereas exclusion of participants with implausible energy intake or diabetes, as well as alternative adiposity specifications, did not materially change the results (Supplementary Tables S1 and S2). Modeling VK intake on the natural log scale strengthened the associations (insulin: −2.6%, HOMA-IR: −2.4%; Supplementary Table S3).

Restricted cubic spline analyses supported an overall association between energy-adjusted VK intake and fasting insulin (*p* < 0.001) and HOMA-IR (*p* = 0.0043). Modest evidence of non-linearity was observed for both fasting insulin (*p* for non-linearity = 0.027) and HOMA-IR (*p* = 0.043). No associations were observed for fasting glucose. In contrast to the linear regression analyses, spline analyses suggested a statistically significant but small non-linear association with HbA1c (overall association *p* < 0.001; *p* for non-linearity = 0.031) (Supplementary Table S5).

## 4. Discussion

### 4.1. Key Findings

In this nationally representative sample of 23,247 U.S. adults, higher energy-adjusted dietary VK intake was modestly associated with lower fasting insulin and HOMA-IR, but not with fasting glucose. Each SD increase in VK intake was associated with 1.4% lower fasting insulin and 1.3% lower HOMA-IR in fully adjusted models. Adjustment for adiposity attenuated approximately half of the association. Effect modification by baseline glycemic status was observed, with stronger associations among normoglycemic participants. The findings were consistent across sensitivity analyses, although effect sizes were small.

While no association was observed for HbA1c in linear models, spline analyses suggested small non-linear associations for fasting insulin, HOMA-IR, and HbA1c; however, the magnitude of these departures from linearity appeared modest and should be interpreted cautiously.

### 4.2. Mechanistic Considerations

Several biological mechanisms have been proposed that may link VK to glucose metabolism [4]. In pancreatic β-cells, VK-dependent regulation of endoplasmic reticulum calcium handling has been shown to preserve glucose-stimulated insulin secretion under metabolic stress [26]; VK may also modulate cAMP-dependent pathways involved in insulin secretion, including Epac2 signaling [25].

In peripheral tissues, animal models demonstrate that VK activates SIRT1-dependent pathways in liver and skeletal muscle, improving insulin signaling and mitochondrial function [32,33]. Beyond direct effects on insulin signaling, VK has been associated with oxidative stress [43,44,45], inflammatory pathways [27,28,29,30,31], and gut microbiota composition, including increased production of butyrate—a metabolite linked to improved insulin sensitivity [46].

Collectively, these findings suggest potential pathways through which VK availability may be associated with insulin signaling and glucose metabolism.

### 4.3. Effect Modification by Baseline Metabolic Status

We observed stronger associations between VK intake and insulin resistance markers in individuals with normoglycemia than in those with impaired glucose metabolism. This pattern differs from findings in randomized controlled trials, where VK supplementation has more consistently improved insulin resistance markers in metabolically impaired individuals [4,38,39,40], with less consistent effects in healthy individuals [4,7,34,35,36,37].

These findings suggest that associations between VK exposure and insulin resistance markers differ across intake ranges and metabolic contexts. Within typical dietary intake levels, they were stronger in individuals with preserved metabolic function. Hypothetically, this may relate to the fact that many of the above-described VK-dependent pathways are extrahepatic and may be only partially activated at usual intake levels [4]. Within this limited range of pathway activation, variation in VK intake may be more strongly associated with insulin sensitivity in individuals with preserved β-cell function and intracellular signaling; however, once metabolic dysfunction becomes established, these associations may be attenuated.

In contrast, pharmacologic and highly bioavailable VK doses used in supplementation trials may allow for fuller activation of extrahepatic VK-dependent pathways [4,7,8,9]. In metabolically healthy individuals—where these pathways already function well—additional activation may yield minimal benefit, while in metabolically impaired states, more pronounced effects on insulin resistance markers may be observed.

This framework may help contextualize differences between observational findings and supplementation trials. However, it remains hypothetical and should be interpreted cautiously because the cross-sectional nature of our findings does not allow inference of causality or determination of whether observed associations reflect biological effects.

### 4.4. Attenuation After Adjustment for Adiposity

Adjustment for BMI and waist circumference attenuated nearly half of the associations, highlighting the complex relationship between adiposity, VK status, and glucose metabolism. While adiposity is a well-established driver of insulin resistance, its relationship with VK status is less clear. As a lipophilic vitamin, VK can be sequestered in adipose tissue, potentially reducing its bioavailability in individuals with higher body fat [14]. Conversely, several population-based analyses have reported inverse associations between VK intake or status and measures of adiposity, particularly visceral fat [13,14,15,16,17].

Adiposity may act as a confounder, a mediator, or both, and these roles cannot be distinguished in a cross-sectional design. Accordingly, these findings should not be interpreted as evidence of specific causal pathways, and the interplay between VK status and adiposity requires further investigation.

### 4.5. Exposure Assessment Considerations

Importantly, dietary VK intake may be a weak proxy for biologically relevant VK exposure. Experimental studies have reported effects on β-cell function and insulin resistance using high-dose, chemically pure VK, whereas a single 24 h recall captures mostly short-term phylloquinone intake and may not reflect habitual intake due to substantial day-to-day variability, nor does it account for the variability in VK absorption, tissue distribution, or conversion to menaquinone-4 [47]. This limitation may introduce measurement error and bias observed associations towards the null.

On the other hand, phylloquinone is abundant in leafy green vegetables and may reflect overall dietary quality. Higher VK intake may therefore reflect healthier dietary patterns rather than independent exposure [47]. Consistent with this, further adjustment for overall diet quality (HEI-2020) attenuated the associations by 24–29% (Supplementary Table S1). While residual confounding by unmeasured aspects of diet cannot be excluded, the associations remained statistically significant after adjustment, suggesting that diet quality alone may not fully account for the observed associations.

### 4.6. Strengths and Limitations

This study has several limitations. First, the cross-sectional design does not allow determination of the direction of observed associations, and reverse causation cannot be excluded. Second, VK intake was assessed using a single 24 h dietary recall, which is subject to measurement error and recall bias. Furthermore, this method may not reflect habitual intake due to substantial day-to-day variability. This variability is likely non-differential and would be expected to attenuate associations toward the null. Third, NHANES does not include biomarkers of VK status, such as circulating phylloquinone or undercarboxylated VK-dependent proteins, which would better capture physiological VK exposure than dietary intake alone [47]. Fourth, although models were adjusted for key determinants of glycemic status, residual confounding by other factors cannot be excluded. Fifth, glycemic outcomes were limited to fasting measures; dynamic indices of insulin sensitivity, which may better reflect extrahepatic insulin resistance, were not available. Finally, the modest magnitude of association warrants careful interpretation of clinical implications.

Despite these limitations, this study has notable strengths. It is, to our knowledge, the first NHANES analysis to directly examine dietary VK intake in relation to insulin resistance markers. The use of a large, nationally representative sample of U.S. adults and standardized laboratory measurements strengthens generalizability. Sequential covariate adjustment, multiple sensitivity analyses, spline modeling, and assessment of effect modification further support the validity of the findings.

## 5. Conclusions

In this nationally representative sample of U.S. adults, higher energy-adjusted dietary VK intake was modestly associated with lower fasting insulin and HOMA-IR, but not with fasting glucose or HbA1c in primary linear models. The stronger associations observed among normoglycemic individuals may reflect differences in associations between VK intake and insulin resistance markers across metabolic states. Further prospective studies incorporating objective assessments of VK status and dynamic measures of insulin sensitivity are needed to clarify these associations.

## Figures and Tables

**Table 1:** Survey-weighted characteristics of study population according to quartiles of energy-adjusted vitamin K intake (NHANES 2001–2018).

Variable	Total (N = 23,247)	Q1 (N = 6055)	Q2 (N = 5905)	Q3 (N = 5755)	Q4 (N = 5531)
Vitamin K intake, µg/1000 kcal	29.4 (18.0, 53.9)	13.0 (9.8, 15.6)	23.2 (20.5, 26.1)	38.7 (33.7, 45.1)	89.5 (67.1, 143.4)
Age, years	46.0 (17.4)	42.1 (16.8)	44.6 (17.1)	47.6 (17.5)	49.5 (17.2)
Sex					
Male	48%	55%	53%	48%	38%
Female	52%	45%	47%	52%	62%
Race/ethnicity					
Mexican American	8.4%	9.1%	11%	8.1%	5.8%
Other Hispanic	5.3%	5.3%	5.5%	5.6%	4.6%
Non-Hispanic White	68%	67%	67%	69%	70%
Non-Hispanic Black	11%	13%	11%	10%	9.9%
Other race	6.9%	5.1%	5.4%	6.9%	10%
Poverty–income ratio	3.0 (1.6)	2.7 (1.6)	2.9 (1.6)	3.0 (1.6)	3.3 (1.6)
Energy intake, kcal/day	2190 (993)	2250 (1103)	2326 (1028)	2215 (943)	1971 (844)
BMI, kg/m^2^	28.8 (6.9)	28.6 (6.8)	29.1 (7.0)	28.9 (6.7)	28.6 (7.0)
Waist circumference, cm	98.3 (16.5)	97.8 (16.5)	99.3 (16.9)	98.8 (16.3)	97.4 (16.3)
Fasting insulin	9.1 (5.9, 14.8)	9.4 (5.9, 15.3)	9.5 (6.2, 15.7)	9.2 (6.0, 15.0)	8.3 (5.4, 13.3)
HOMA-IR	2.3 (1.4, 3.9)	2.3 (1.4, 4.0)	2.4 (1.5, 4.1)	2.3 (1.4, 4.0)	2.0 (1.3, 3.5)
Fasting glucose, mg/dL	104.7 (29.4)	103.1 (29.3)	105.0 (28.9)	105.9 (30.5)	104.9 (28.9)
HbA1c, %	5.6 (0.9)	5.5 (0.9)	5.6 (0.9)	5.6 (0.9)	5.6 (0.9)
Smoking status					
Current smoker	21%	30%	21%	18%	14%
Non-smoker	79%	70%	79%	82%	86%
Alcohol use					
Drinker	78%	78%	77%	78%	77%
Non-drinker	22%	22%	23%	22%	23%
Diabetes	12%	11%	12%	13%	14%

Notes: Values are survey-weighted means ± standard deviations (SDs) for continuous variables and weighted percentages for categorical variables, except for energy-adjusted vitamin K intake, insulin, and HOMA-IR, which are presented as medians (interquartile ranges). Sample sizes (N) are unweighted. Quartiles were defined using survey-weighted cutpoints of energy-adjusted vitamin K intake (µg/1000 kcal). Abbreviations: BMI, body mass index; HbA1c, glycated hemoglobin.

**Table 2:** Associations between quartiles of energy-adjusted vitamin K intake and glycemic markers.

Model/Quartile	Insulin (% Difference)	HOMA-IR (% Difference)	Glucose (mg/dL)	HbA1c (%)
Model 1: unadjusted				
Q2 vs. Q1	3.8% (−0.6, 8.3)	5.8% (0.9, 10.8) *	1.85 (0.56, 3.14) *	0.07 (0.03, 0.10) *
Q3 vs. Q1	0.3% (−3.2, 3.9)	2.9% (−1.1, 6.9)	2.74 (1.39, 4.09) *	0.10 (0.06, 0.14) *
Q4 vs. Q1	−8.7% (−12.1, −5.2) *	−7.0% (−10.9, −3.0) *	1.81 (0.44, 3.19) *	0.09 (0.05, 0.13) *
Model 2: lifestyle-adjusted				
Q2 vs. Q1	1.3% (−3.2, 6.1)	2.1% (−2.9, 7.4)	0.64 (−0.79, 2.08)	0.03 (−0.01, 0.08)
Q3 vs. Q1	−0.6% (−4.3, 3.2)	0.1% (−3.9, 4.3)	0.75 (−0.67, 2.18)	0.03 (−0.01, 0.08)
Q4 vs. Q1	−8.4% (−12.0, −4.6) *	−8.1% (−12.2, −3.8) *	0.15 (−1.39, 1.69)	0.02 (−0.03, 0.06)
Model 3: adiposity-adjusted				
Q2 vs. Q1	−0.8% (−4.3, 2.7)	−0.3% (−4.0, 3.5)	0.32 (−1.12, 1.75)	0.02 (−0.02, 0.06)
Q3 vs. Q1	−0.4% (−3.3, 2.6)	0.4% (−2.7, 3.7)	0.85 (−0.57, 2.27)	0.04 (−0.01, 0.08)
Q4 vs. Q1	−6.6% (−9.5, −3.6) *	−6.2% (−9.4, −3.0) *	0.16 (−1.29, 1.61)	0.02 (−0.02, 0.06)
*p* for trend (model 3)	<0.001 *	<0.001 *	0.971	0.681

Notes: Values are survey-weighted linear regression coefficients (β) with 95% confidence intervals. β represents the difference in glycemic measures compared with the lowest quartile (Q1) of energy-adjusted vitamin K intake. Insulin and HOMA-IR estimates represent percent differences derived from log-transformed models. *p* for trend was calculated by modeling quartile medians as an ordinal variable in Model 3. *p*-values < 0.05 are denoted by an *. Abbreviations: HbA1c, glycated hemoglobin; HOMA-IR, homeostasis model assessment of insulin resistance.

**Table 3:** Associations between energy-adjusted vitamin K intake (per SD increase) and glycemic markers (Model 3).

Outcome	β (95% CI)	*p*-Value
Insulin, % difference	−1.4% (−2.4, −0.4)	0.0067 *
HOMA-IR, % difference	−1.3% (−2.3, −0.3)	0.014 *
Glucose, mg/dL	0.13 (−0.16, 0.43)	0.365
HbA1c, %	0.00 (−0.02, 0.01)	0.740

Notes: Values are survey-weighted linear regression coefficients (β) with 95% CIs from fully adjusted models (Model 3). β represents the change in glycemic measures per SD increase in energy-adjusted vitamin K intake. Insulin and HOMA-IR estimates represent percent differences derived from log-transformed models. *p*-values < 0.05 are denoted by an *. Abbreviations: CI, confidence interval; HbA1c, glycated hemoglobin; HOMA-IR, homeostasis model assessment of insulin resistance.

**Table 4:** Effect modification of the association between energy-adjusted vitamin K intake and insulin resistance by baseline metabolic status.

Baseline Metabolic Status	Fasting Insulin (% Difference)	HOMA-IR (% Difference)
Normoglycemic (N = 6925)	−3.4% (−5.2, −1.6) *	−3.4% (−5.2, −1.5) *
Impaired (prediabetes) (N = 6892)	−0.8% (−1.2, −0.3) *	−0.7% (−1.1, −0.2) *
*p* for interaction	0.0052 *	0.0062 *

Notes: Impaired glucose metabolism (prediabetes) was defined as a fasting glucose level of 100–125 mg/dL or an HbA1c of 5.7–6.4%. Analyses were restricted to participants without diabetes. Unweighted N represents the analyzed (complete-case) sample size for this model. Values are survey-weighted linear regression coefficients (β) with 95% confidence intervals from fully adjusted models (Model 3). β represents the percent difference in insulin and HOMA-IR per 1 SD increase in energy-adjusted vitamin K intake (µg/1000 kcal), derived from log-transformed models. *p* for interaction derived from multiplicative interaction terms. *p*-values < 0.05 are denoted by an *. Abbreviations: HOMA-IR, homeostasis model assessment of insulin resistance.

## Data Availability

The data analyzed in this study are publicly available from the National Health and Nutrition Examination Survey (NHANES) conducted by the U.S. National Center for Health Statistics (NCHS) of the Centers for Disease Control and Prevention (CDC) and can be accessed at https://www.cdc.gov/nchs/nhanes/ (accessed on 4 May 2026). The full R code used for data cleaning, statistical analyses, and table generation is publicly available at: https://github.com/Mikolaj-Madeksza/nhanes-vitamin-k-glycemic-markers/ (accessed on 4 May 2026).

## Supplementary Materials

The following supporting information can be downloaded at https://www.mdpi.com/article/10.3390/jcm15103763/s1, Figure S1. Directed acyclic graph (DAG) illustrating conceptual relationships between vitamin K status and glycemic markers; Figure S2. Prespecified analytical strategy for evaluating associations between energy-adjusted vitamin K intake and glycemic markers; Table S1. Sensitivity analyses of the association between energy-adjusted vitamin K intake (per SD) and fasting insulin and HOMA-IR; Table S2. Sensitivity analysis using alternative adiposity specifications; Table S3. Association between log-transformed energy-adjusted vitamin K intake and glycemic markers (Model 3); Table S4. Percent attenuation of associations between energy-adjusted vitamin K intake and fasting insulin and HOMA-IR across sequential adjustment models; Table S5. Survey-weighted restricted cubic spline analysis of energy-adjusted vitamin K intake and glycemic markers; Table S6. Effect modification of associations between energy-adjusted vitamin K intake and fasting insulin and HOMA-IR by sex and obesity status.

## Abbreviations

The following abbreviations are used in this manuscript:

NHANES	National Health and Nutrition Examination Survey
HOMA-IR	Homeostatic Model Assessment—Insulin Resistance
DM2	Type 2 diabetes
VK	Vitamin K

## References

[1] Ho H.-J., Komai M., Shirakawa H. (2020). Beneficial Effects of Vitamin K Status on Glycemic Regulation and Diabetes Mellitus: A Mini-Review. Nutrients.

[2] Karamzad N., Maleki V., Carson-Chahhoud K., Azizi S., Sahebkar A., Gargari B.P. (2020). A Systematic Review on the Mechanisms of Vitamin K Effects on the Complications of Diabetes and Pre-diabetes. BioFactors.

[3] Tan J., Li Y. (2024). Revisiting the Interconnection between Lipids and Vitamin K Metabolism: Insights from Recent Research and Potential Therapeutic Implications: A Review. Nutr. Metab..

[4] Matuszewski W., Madeksza M., Szklarz M., Rutkiewicz A., Rutkowska J., Harazny J.M. (2026). Vitamin K as an Endocrine Modulator: Mechanistic Links to Glucose Metabolism and Beyond. Nutrients.

[5] Kaźmierczak-Barańska J., Karwowski B.T. (2022). Vitamin K Contribution to DNA Damage—Advantage or Disadvantage? A Human Health Response. Nutrients.

[6] Lee M.H., Cho Y., Kim D.H., Woo H.J., Yang J.Y., Kwon H.J., Yeon M.J., Park M., Kim S.-H., Moon C. (2016). Menadione Induces G2/M Arrest in Gastric Cancer Cells by down-Regulation of CDC25C and Proteasome Mediated Degradation of CDK1 and Cyclin B1. Am. J. Transl. Res..

[7] Pan Y., Jackson R.T. (2009). Dietary Phylloquinone Intakes and Metabolic Syndrome in US Young Adults. J. Am. Coll. Nutr..

[8] McCann J.C., Ames B.N. (2009). Vitamin K, an Example of Triage Theory: Is Micronutrient Inadequacy Linked to Diseases of Aging?. Am. J. Clin. Nutr..

[9] Vermeer C.V. (2012). Vitamin K: The Effect on Health beyond Coagulation—An Overview. Food Nutr. Res..

[10] Zwakenberg S.R., Remmelzwaal S., Beulens J.W.J., Booth S.L., Burgess S., Dashti H.S., Imamura F., Feskens E.J.M., Van Der Schouw Y.T., Sluijs I. (2019). Circulating Phylloquinone Concentrations and Risk of Type 2 Diabetes: A Mendelian Randomization Study. Diabetes.

[11] Beulens J.W.J., Van Der A D.L., Grobbee D.E., Sluijs I., Spijkerman A.M.W., Van Der Schouw Y.T. (2010). Dietary Phylloquinone and Menaquinones Intakes and Risk of Type 2 Diabetes. Diabetes Care.

[12] Ibarrola-Jurado N., Salas-Salvadó J., Martínez-González M.A., Bulló M. (2012). Dietary Phylloquinone Intake and Risk of Type 2 Diabetes in Elderly Subjects at High Risk of Cardiovascular Disease. Am. J. Clin. Nutr..

[13] Dam V., Dalmeijer G.W., Vermeer C., Drummen N.E., Knapen M.H., Van Der Schouw Y.T., Beulens J.W. (2015). Association Between Vitamin K and the Metabolic Syndrome: A 10-Year Follow-Up Study in Adults. J. Clin. Endocrinol. Metab..

[14] Shea M.K., Booth S.L., Gundberg C.M., Peterson J.W., Waddell C., Dawson-Hughes B., Saltzman E. (2010). Adulthood Obesity Is Positively Associated with Adipose Tissue Concentrations of Vitamin K and Inversely Associated with Circulating Indicators of Vitamin K Status in Men and Women. J. Nutr..

[15] Knapen M.H.J., Schurgers L.J., Shearer M.J., Newman P., Theuwissen E., Vermeer C. (2012). Association of Vitamin K Status with Adiponectin and Body Composition in Healthy Subjects: Uncarboxylated Osteocalcin Is Not Associated with Fat Mass and Body Weight. Br. J. Nutr..

[16] Villa T.H.C., Ruiz-Vivanco G., Porchia L.M., Torres-Rasgado E., López-Bayghen E., Gonzalez-Mejia M.E. (2026). Dietary Vitamins A and K Are Inversely Associated with Visceral Adiposity in US Adults: NHANES 2011–2018. Nutr. Res..

[17] Ravera M., Nickolas T., Plebani M., Iervasi G., Aghi A., Khairallah P., Gallieni M., Mereu M.C., Giannini S., Sella S. (2021). Overweight-Obesity Is Associated with Decreased Vitamin K2 Levels in Hemodialysis Patients. Clin. Chem. Lab. Med..

[18] Shea M.K., Booth S.L., Massaro J.M., Jacques P.F., D’Agostino R.B., Dawson-Hughes B., Ordovas J.M., O’Donnell C.J., Kathiresan S., Keaney J.F. (2008). Vitamin K and Vitamin D Status: Associations with Inflammatory Markers in the Framingham Offspring Study. Am. J. Epidemiol..

[19] Shea S., O’Donnell H.J., Vermeer C., Magdeleyns E.J.P., Crosier M.D., Gundberg C.M., Ordovas J.M., Kritchevsky S.B., Booth B. (2011). Circulating Uncarboxylated Matrix Gla Protein Is Associated with Vitamin K Nutritional Status, but Not Coronary Artery Calcium, in Older Adults. J. Nutr..

[20] Brnic D., Martinovic D., Zivkovic P.M., Tokic D., Vilovic M., Rusic D., Hadjina I.T., Libers C., Glumac S., Supe-Domic D. (2020). Inactive Matrix Gla Protein Is Elevated in Patients with Inflammatory Bowel Disease. World J. Gastroenterol..

[21] Roumeliotis S., Roumeliotis A., Stamou A., Leivaditis K., Kantartzi K., Panagoutsos S., Liakopoulos V. (2020). The Association of Dp-ucMGP with Cardiovascular Morbidity and Decreased Renal Function in Diabetic Chronic Kidney Disease. Int. J. Mol. Sci..

[22] Schweighofer N., Colantonio C., Haudum C.W., Hutz B., Kolesnik E., Schmidt A., Zirlik A., Pieber T.R., Verheyen N., Obermayer-Pietsch B. (2022). Is MGP an Inflammatory Marker?. Endocr. Abstr..

[23] Luo W., Ye D., Zhao K., Zhou L., Wu Y., Ge Q. (2025). Associations between Vitamin K and Systemic Immune and Inflammation Biomarkers: A Population-Based Study from the NHANES (2007–2020). Front. Nutr..

[24] Wang F., Sun M., Guo R., Wu Z., Wang X., Yang Y., Liu Y., Dong Y., Wang S., Yan S. (2024). The Association between Vitamin K Intake and Dyslipidemia in US Adults: The Mediating Effect of Insulin Resistance. Food Funct..

[25] Ho H.-J., Shirakawa H., Hirahara K., Sone H., Kamiyama S., Komai M. (2019). Menaquinone-4 Amplified Glucose-Stimulated Insulin Secretion in Isolated Mouse Pancreatic Islets and INS-1 Rat Insulinoma Cells. Int. J. Mol. Sci..

[26] Lacombe J., Guo K., Bonneau J., Faubert D., Gioanni F., Vivoli A., Muir S.M., Hezzaz S., Poitout V., Ferron M. (2023). Vitamin K-Dependent Carboxylation Regulates Ca2+ Flux and Adaptation to Metabolic Stress in β Cells. Cell Rep..

[27] Staudinger J.L., Mahroke A., Patel G., Dattel C., Reddy S. (2024). Pregnane X Receptor Signaling Pathway and Vitamin K: Molecular Mechanisms and Clinical Relevance in Human Health. Cells.

[28] Kieronska-Rudek A., Kij A., Kaczara P., Tworzydlo A., Napiorkowski M., Sidoryk K., Chlopicki S. (2021). Exogenous Vitamins K Exert Anti-Inflammatory Effects Dissociated from Their Role as Substrates for Synthesis of Endogenous MK-4 in Murine Macrophages Cell Line. Cells.

[29] Dihingia A., Ozah D., Baruah P.K., Kalita J., Manna P. (2018). Prophylactic Role of Vitamin K Supplementation on Vascular Inflammation in Type 2 Diabetes by Regulating the NF-κB/Nrf2 Pathway via Activating Gla Proteins. Food Funct..

[30] Van Der Meer J.H.M., Van Der Poll T., Van ‘T Veer C. (2014). TAM Receptors, Gas6, and Protein S: Roles in Inflammation and Hemostasis. Blood.

[31] Li J., Wang H., Rosenberg P.A. (2009). Vitamin K Prevents Oxidative Cell Death by Inhibiting Activation of 12-lipoxygenase in Developing Oligodendrocytes. J. Neurosci. Res..

[32] Dihingia A., Ozah D., Ghosh S., Sarkar A., Baruah P.K., Kalita J., Sil P.C., Manna P. (2018). Vitamin K1 Inversely Correlates with Glycemia and Insulin Resistance in Patients with Type 2 Diabetes (T2D) and Positively Regulates SIRT1/AMPK Pathway of Glucose Metabolism in Liver of T2D Mice and Hepatocytes Cultured in High Glucose. J. Nutr. Biochem..

[33] Su X., Wang W., Fang C., Ni C., Zhou J., Wang X., Zhang L., Xu X., Cao R., Lang H. (2021). Vitamin K2 Alleviates Insulin Resistance in Skeletal Muscle by Improving Mitochondrial Function via SIRT1 Signaling. Antioxid. Redox Signal..

[34] Centi A.J. (2015). Association of Vitamin K with Insulin Resistance and Body Composition. Ph.D. Dissertation.

[35] Kumar R., Binkley N., Vella A. (2010). Effect of Phylloquinone Supplementation on Glucose Homeostasis in Humans. Am. J. Clin. Nutr..

[36] Knapen M.H.J., Braam L.A.J.L.M., Drummen N.E., Bekers O., Hoeks A.P.G., Vermeer C. (2015). Menaquinone-7 Supplementation Improves Arterial Stiffness in Healthy Postmenopausal Women: A Double-Blind Randomised Clinical Trial. Thromb. Haemost..

[37] Shahdadian F., Mohammadi H., Rouhani M. (2018). Effect of Vitamin K Supplementation on Glycemic Control: A Systematic Review and Meta-Analysis of Clinical Trials. Horm. Metab. Res..

[38] Khatapoush S., Mohit M., Mansouri Shirazi F., Moazen M., Ebrahimzadeh A., Hejazi N. (2025). The Effect of Vitamin K Supplementation on Glycemic Indices in Adults: A Systematic Review and Meta- Analysis of Clinical Trials. Int. J. Nutr. Sci..

[39] Nikpayam O., Jafari A., Faghfouri A., Pasand M., Noura P., Najafi M., Sohrab G. (2025). Effect of Menaquinone-7 (MK-7) Supplementation on Anthropometric Measurements, Glycemic Indices, and Lipid Profiles: A Systematic Review and Meta-Analysis of Randomized Controlled Trials. Prostaglandins Other Lipid Mediat..

[40] Karamali M., Ashrafi M., Razavi M., Jamilian M., Akbari M., Asemi Z. (2017). The Effects of Calcium, Vitamins D and K Co-Supplementation on Markers of Insulin Metabolism and Lipid Profiles in Vitamin D-Deficient Women with Polycystic Ovary Syndrome. Exp. Clin. Endocrinol. Diabetes Off. J. Ger. Soc. Endocrinol. Ger. Diabetes Assoc..

[41] Centers for Disease Control and Prevention NHANES NCHS Ethics Review Board Approval 1999–2022. https://www.cdc.gov/nchs/nhanes/about/erb.html.

[42] Centers for Disease Control and Prevention NHANES Survey Methods and Analytic Guidelines. https://wwwn.cdc.gov/nchs/nhanes/analyticguidelines.aspx.

[43] Wu J., Shi Y., Zhou M., Chen M., Ji S., Liu X., Zhou M., Xia R., Zheng X., Wang W. (2024). Nutrient Vitamins Enabled Metabolic Regulation of Ferroptosis via Reactive Oxygen Species Biology. Front. Pharmacol..

[44] Mishima E., Ito J., Wu Z., Nakamura T., Wahida A., Doll S., Tonnus W., Nepachalovich P., Eggenhofer E., Aldrovandi M. (2022). A Non-Canonical Vitamin K Cycle Is a Potent Ferroptosis Suppressor. Nature.

[45] Kolbrink B., Von Samson-Himmelstjerna F.A., Messtorff M.L., Riebeling T., Nische R., Schmitz J., Bräsen J.H., Kunzendorf U., Krautwald S. (2022). Vitamin K1 Inhibits Ferroptosis and Counteracts a Detrimental Effect of Phenprocoumon in Experimental Acute Kidney Injury. Cell. Mol. Life Sci..

[46] Mathers J.C., Fernandez F., Hill M.J., McCarthy P.T., Shearer M.J., Oxley A. (1990). Dietary Modification of Potential Vitamin K Supply from Enteric Bacterial Menaquinones in Rats. Br. J. Nutr..

[47] Shea M., Booth S. (2016). Concepts and Controversies in Evaluating Vitamin K Status in Population-Based Studies. Nutrients.

