# Supplementary material for: Dietary Vitamin K Intake and Insulin Resistance Markers in U.S. Adults: NHANES 2001–2018
Source: J Clin Med. 2026 May 14;15(10):3763. doi: 10.3390/jcm15103763 (PMC13207640; doi:10.3390/jcm15103763)
Supplement: Supplementary file 1 [file jcm-15-03763-s001.zip › jcm-4243850-supplementary.pdf]

Supplementary information

Supplementary Figure S1. Directed acyclic graph (DAG) illustrating conceptual relationships between vitamin K status and glycemic markers

Supplementary Figure S2. Prespecified analytical strategy for evaluating associations between energy-adjusted vitamin K intake and glycemic markers

Supplementary Table S1. Sensitivity analyses of the association between energy-adjusted vitamin K intake (per SD) and fasting insulin and HOMA-IR

Supplementary Table S2. Sensitivity analysis using alternative adiposity specifications

Supplementary Table S3. Association between log-transformed energy-adjusted vitamin K intake and glycemic markers (Model 3)

Supplementary Table S4. Percent attenuation of associations between energy-adjusted vitamin K intake and fasting insulin and HOMA-IR across sequential adjustment models

Supplementary Table S5. Survey-weighted restricted cubic spline analysis of energy-adjusted vitamin K intake and glycemic markers

Supplementary Table S6. Effect modification of associations between energy-adjusted vitamin K intake and fasting insulin and HOMA-IR by sex and obesity status

Supplementary Figure S1. Directed acyclic graph (DAG) illustrating conceptual relationships between vitamin K status and glycemic markers

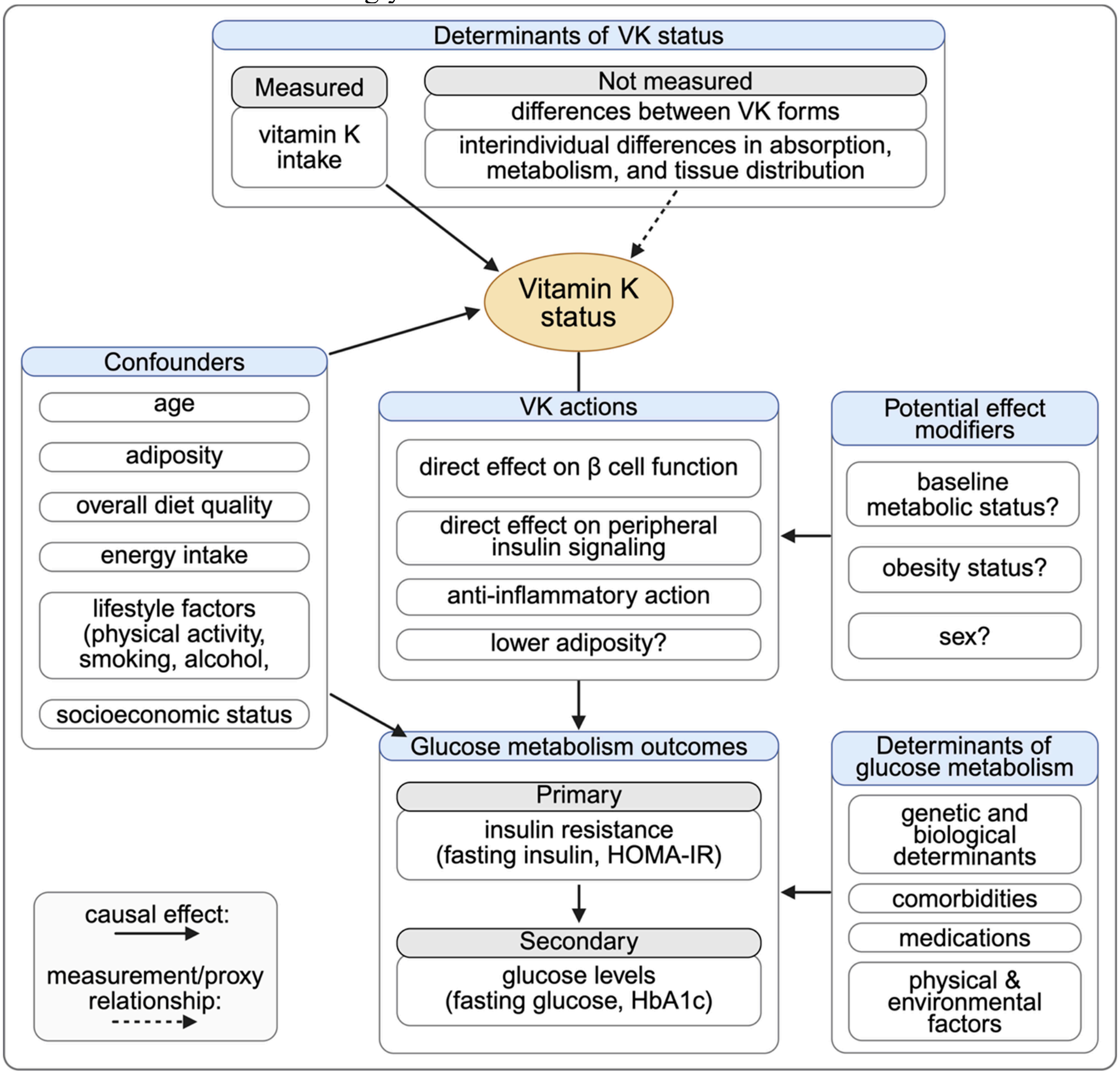

Notes: The DAG depicts hypothesized relationships among vitamin K intake, vitamin K status, potential confounders, mediators, and glucose metabolism outcomes. Confounders include factors that plausibly influence both vitamin K and glucose metabolism status.
Dashed arrow indicates that the observed variable represents, but does not fully capture, underlying biological vitamin K status.
Abbreviations: DAG, directed acyclic graph; HbA1c, glycated hemoglobin; HOMA-IR, homeostasis model assessment of insulin resistance; VK, vitamin K.

Supplementary Figure S2. Prespecified analytical strategy for evaluating associations between energy-adjusted vitamin K intake and glycemic outcomes

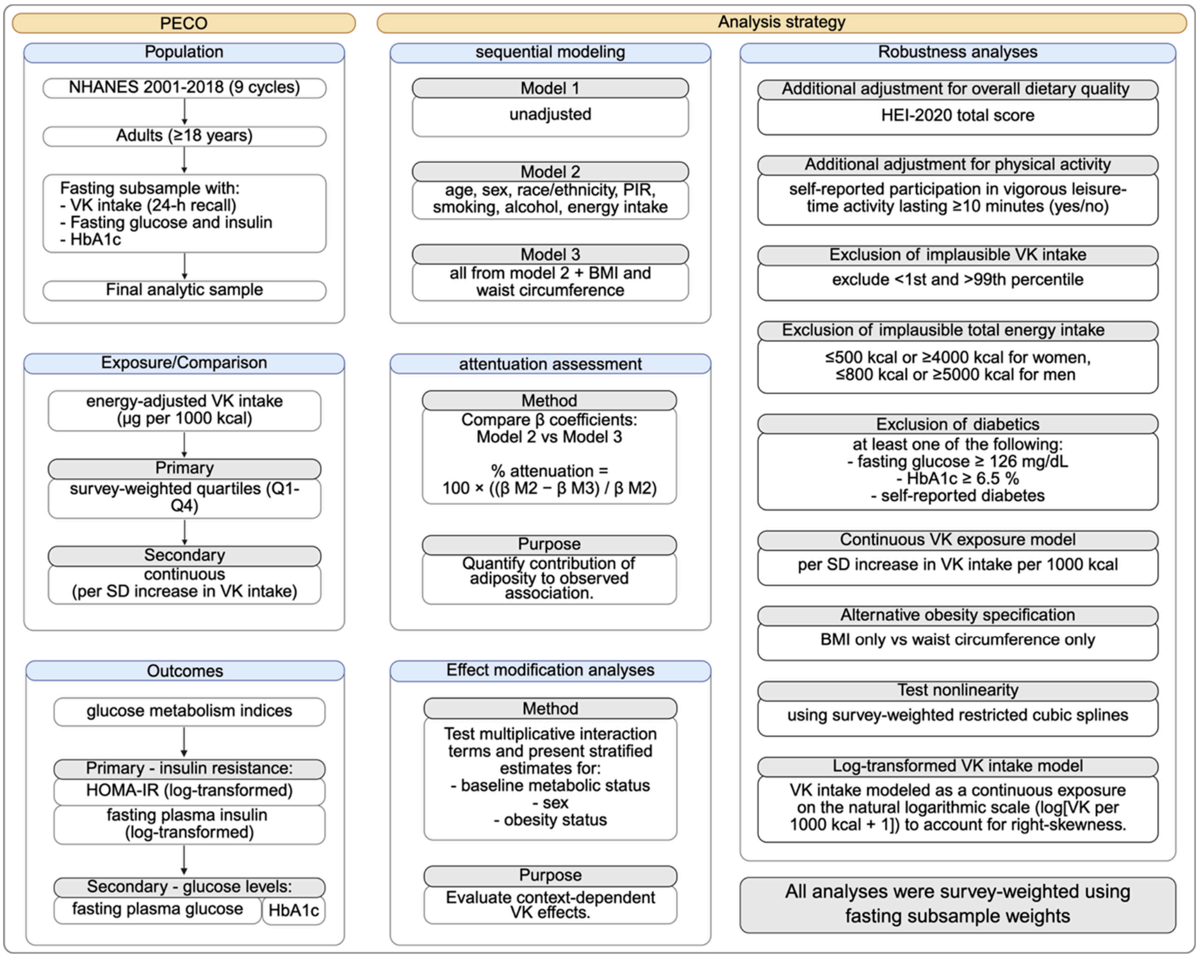

Notes: The diagram summarizes the selection of study population, exposure, and outcomes (PECO), and the full prespecified analysis plan, including primary and robustness analyses, and effect modification analyses. Abbreviations: BMI, body mass index; HbA1c, glycated hemoglobin; HEI-2020, Healthy Eating Index-2020; HOMA-IR, homeostasis model assessment of insulin resistance; SD, standard deviation; VK, vitamin K.

Supplementary Table S1. Sensitivity analyses of the association between energy-adjusted vitamin K intake (per SD) and fasting insulin and HOMA-IR

Model/ outcome	N (unweighted)	β (95% CI)	P-value	% change vs. primary
Model 3 (primary)				
Fasting insulin	16546	-1.4 (-2.4, -0.4)	0.007*	-
HOMA-IR	16544	-1.3 (-2.3, -0.3)	0.014*	-
Additional adjustment for overall diet quality (HEI-2020)				
Fasting insulin	13071	-1.1 (-1.9, -0.3)	0.011*	-24.6%
HOMA-IR	13071	-0.9 (-1.8, -0.1)	0.031*	-28.2%
Additional adjustment for physical activity				
Fasting insulin	16360	-1.3 (-2.2, -0.4)	0.004*	-9.2%
HOMA-IR	16358	-1.2 (-2.0, -0.3)	0.010*	-11.5%
Excluding extreme VK intake (1st-99th percentile)				
Fasting insulin	16242	-2.3 (-3.4, -1.1)	<0.001*	+61.3%
HOMA-IR	16240	-2.2 (-3.5, -1.0)	<0.001*	+70.2%
Excluding implausible energy intake				
Fasting insulin	15988	-1.4 (-2.5, -0.4)	0.008*	+0.7%
HOMA-IR	15987	-1.3 (-2.4, -0.3)	0.016*	+2.3%
Excluding participants with diabetes				
Fasting insulin	13817	-1.3 (-2.2, -0.3)	0.009*	-9.9%
HOMA-IR	13817	-1.2 (-2.2, -0.2)	0.022*	-9.9%

Notes: Values are survey-weighted linear regression coefficients (β) with 95% confidence intervals.
β represents the percent difference in insulin and HOMA-IR per 1 SD increase in energy-adjusted vitamin K intake (μg/1000 kcal), derived from log-transformed models.
The primary model (Model 3) is adjusted for age, sex, race/ethnicity, poverty-income ratio, smoking status, alcohol use, total energy intake, body mass index, and waist circumference.
Sensitivity models were based on Model 3 and additionally incorporated the covariate adjustments or exclusions specified in the table.
Physical activity was defined as self-reported participation in any vigorous leisure-time activity lasting ≥10 minutes (yes/no).
Implausible energy intake was defined as total energy intake of ≤ 500 kcal or ≥ 4000 kcal for women and ≤ 800 kcal or ≥ 5000 kcal for men; diabetes was defined as fasting glucose ≥ 126 mg/dl, HbA1c ≥ 6.5 %, or self-reported diabetes diagnosed by doctor.
Percent change vs. primary reflects the relative change in β compared with the primary fully adjusted model (Model 3).
Unweighted N represents the analyzed (complete-case) sample size for each model.
P-values <0.05 are denoted by (*).
Abbreviations: CI, confidence interval; HEI-2020; Healthy Eating Index-2020; HOMA-IR, homeostasis model assessment of insulin resistance; SD, standard deviation.

Supplementary Table S2. Sensitivity analysis using alternative adiposity specifications

Model/outcome	N (unweighted)	β (95% CI)	P-value	% change vs. primary
Original Model 3 (BMI + WC)				
Fasting insulin	16546	-1.4 (-2.4, -0.4)	0.007*	-
HOMA-IR	16544	-1.3 (-2.3, -0.3)	0.014*	-
BMI adjustment only				
Fasting insulin	16793	-1.5 (-2.6, -0.4)	0.007*	+8.5%
HOMA-IR	16791	-1.4 (-2.5, -0.3)	0.012*	+9.2%
WC adjustment only				
Fasting insulin	16611	-1.4 (-2.5, -0.4)	0.007*	+2.1%
HOMA-IR	16609	-1.3 (-2.4, -0.3)	0.013*	+2.3%

Notes: Values are survey-weighted linear regression coefficients (β) with 95% confidence intervals.
β represents the percent difference in insulin and HOMA-IR per 1 SD increase in energy-adjusted vitamin K intake (μg/1000 kcal), derived from log-transformed models.
The primary adiposity-adjusted model (Model 3) is based on Model 2 and additionally adjusts for BMI and WC. Sensitivity analyses compare this primary model with models including BMI alone or WC alone to evaluate the relative contribution of each adiposity measure to attenuation of the association.
Percent change vs. primary reflects the relative change in β compared with the primary model (BMI + WC).
P-values <0.05 are denoted by (*).
Abbreviations: BMI, body mass index; HOMA-IR, homeostasis model assessment of insulin resistance; WC, waist circumference.

Supplementary Table S3. Association between log-transformed energy-adjusted vitamin K intake and glycemic markers (Model 3)

Outcome	β (95% CI)	P value
Insulin, % difference	-2.6% (-3.8, -1.3)	<0.001*
HOMA-IR, % difference	-2.4% (-3.8, -1.0)	<0.001*
Glucose, mg/dL	0.12 (-0.44, 0.68)	0.666
HbA1c, %	0.01 (-0.01, 0.03)	0.181

Notes: Values are survey-weighted linear regression coefficients (β) with 95% CI from fully adjusted models (Model 3).

β represents the change in glycemic measures per SD increase in natural log-transformed energy-adjusted vitamin K intake.

Insulin and HOMA-IR estimates represent percent differences derived from log-transformed models.

P-values < 0.05 are denoted by (*).

Abbreviations: CI, confidence intervals; HbA1c, glycated hemoglobin; HOMA-IR, homeostasis model assessment of insulin resistance.

Supplementary Table S4. Percent attenuation of associations between energy-adjusted vitamin K intake and fasting insulin and HOMA-IR across sequential adjustment models

Outcome	Attenuation M1 to M2 (%)	Attenuation M2 to M3 (%)	Total attenuation M1 to M3 (%)
Fasting insulin	18.6	44.2	54.6
HOMA-IR	13.6	48.5	55.5

Notes: Percent attenuation was calculated as the proportional change in the regression coefficient (β) between sequential models: [Percent attenuation = ((β earlier model - β later model)/β earlier model) × 100].
Positive values indicate attenuation toward the null.
Abbreviations: HOMA-IR, homeostasis model assessment of insulin resistance.

Supplementary Table S5. Survey-weighted restricted cubic spline analysis of energy-adjusted vitamin K intake and glycemic markers

Outcome	P overall spline association	P for non-linearity
Insulin (% difference)	<0.001*	0.027*
HOMA-IR (% difference)	0.0043*	0.043*
Glucose (mg/dL)	0.296	0.915
HbA1c (%)	<0.001*	0.031*

Notes: P-values derived from survey-weighted restricted cubic spline models adjusted as in Model 3.
P-values <0.05 are denoted by (*).
Abbreviations: HbA1c, glycated hemoglobin; HOMA-IR, homeostasis model assessment of insulin resistance.

Supplementary Table S6. Effect modification of associations between energy-adjusted vitamin K intake and fasting insulin and HOMA-IR by sex and obesity status

Modifier/group	Fasting insulin (% difference)	HOMA-IR (% difference)
Sex		
Female	-1.4% (-2.8, 0.0)	-1.2% (-2.8, 0.5)
Male	-1.4% (-2.8, 0.0)	-1.4% (-2.8, 0.1)
**P for interaction**	0.960	0.849
Obesity		
Non-obese	-1.7% (-2.9, -0.4)	-1.6% (-2.9, -0.3)
Obese	-1.8% (-4.0, 0.4)	-1.5% (-4.0, 1.1)
**P for interaction**	0.888	0.917

Notes: Obesity was defined as BMI ≥30 kg/m^2.
Values are survey-weighted linear regression coefficients (β) with 95% confidence intervals.
β represents the percent difference in insulin and HOMA-IR per 1 SD increase in energy-adjusted vitamin K intake (µg/1000 kcal), derived from log-transformed models.
Sex-stratified model was fully adjusted (Model 3), while obesity-stratified model did not adjust for adiposity (Model 2).
P for interaction is derived from multiplicative interaction terms.
Abbreviations: BMI, body mass index; HOMA-IR, homeostasis model assessment of insulin resistance.
